# Galactorrhea during antipsychotic treatment: results from AMSP, a drug surveillance program, between 1993 and 2015

**DOI:** 10.1007/s00406-021-01241-3

**Published:** 2021-03-25

**Authors:** C. Glocker, R. Grohmann, R. Engel, J. Seifert, S. Bleich, S. Stübner, S. Toto, C. Schüle

**Affiliations:** 1 Department of Psychiatry und Psychotherapy, Klinik für Psychiatrie und Psychotherapie der LMU München, LMU Klinikum, Nußbaumstraße 7, 80336 Munich, Germany; 2 Department of Psychiatry, Social Psychiatry and Psychotherapy, Hannover Medical School, Carl-Neuberg-Str. 1, 30625 Hannover, Germany; 3 Department of Forensic Psychiatry, Bezirksklinikum Ansbach, Feuchtwanger Str. 38, 91522 Ansbach, Germany

**Keywords:** AMSP program, Galactorrhea, Antipsychotic drugs, Adverse drug reactions, Psychiatric inpatients

## Abstract

Galactorrhea is a well-known adverse drug reaction (ADR) of numerous antipsychotic drugs (APD) and is often distressing for those affected. Methodological problems in the existing literature make it difficult to determine the prevalence of symptomatic hyperprolactinemia in persons treated with APDs. Consequently, a large sample of patients exposed to APDs is needed for more extensive evaluation. Data on APD utilization and reports of galactorrhea caused by APDs were analyzed using data from an observational pharmacovigilance program in German-speaking countries—Arzneimittelsicherheit in der Psychiatrie (AMSP)—from 1993 to 2015. 320,383 patients (175,884 female inpatients) under surveillance were treated with APDs for schizophrenia and other indications. A total of 170 events of galactorrhea caused by APDs were identified (0.97 cases in 1000 female inpatient admissions). Most cases occurred during the reproductive age with the highest incidence among patients between 16 and 30 years (3.81 cases in 1000 inpatients). The APDs that were most frequently imputed alone for inducing galactorrhea were risperidone (52 cases and 0.19% of all exposed inpatients), amisulpride (30 resp. 0.48%), and olanzapine (13 resp. 0.05%). In three cases, quetiapine had a prominent role as a probable cause for galactorrhea. High dosages of the imputed APDs correlated with higher rates of galactorrhea. Galactorrhea is a severe and underestimated condition in psychopharmacology. While some APDs are more likely to cause galactorrhea, we identified a few unusual cases. This highlights the importance of alertness in clinical practice and of taking a patient’s individual situation into consideration.

## Introduction

Blockage of dopaminergic D_2_ receptors in mesolimbic and mesocortical pathways of the brain is an active principle that is implemented to varying degrees in most antipsychotic drugs (APD). In other areas of the brain, however, blockade of D_2_ receptors can cause significant adverse effects. Extrapyramidal side effects such as Parkinsonism result from D_2_ antagonism in the striatum, while blockage of D_2_ receptors on lactotroph cells causes hyperprolactinemia due to removal of the main inhibitory influence (i.e., dopamine) [1].

A cohort study from 2017 reported that among 194 patients with schizophrenia and bipolar disorder receiving APDs, 38% had prolactin levels above the upper limit of normal, two-thirds of whom had significantly elevated levels with clinical presentation. Women were particularly at risk for elevated prolactin levels and associated manifestations, with over 50% of women in the study having abnormal prolactin levels [2].

Studies with first-generation APDs demonstrate that therapeutic doses of any of the applied drugs may cause up to tenfold prolactin elevations which have been reported to correlate to dose [3]. It seems that the faster the APD dissociates from D_2_ receptors, the lesser the increase of prolactin in plasma. The role of their active metabolites should also be considered. Accordingly, APDs can be roughly distinguished as “prolactin-raising” (conventional neuroleptics, amisulpride, risperidone, and paliperidone) and “prolactin-sparing” (clozapine, aripiprazole, olanzapine, quetiapine, and ziprasidone) APDs [1, 4]. Methodological problems in the existing literature make it difficult to determine the prevalence of symptomatic hyperprolactinemia in persons treated with APDs. Many previous studies are limited to the sole assessment of prolactin levels and not their clinical manifestations. Due to inter-individual differences in response, elevated prolactin may occasionally be asymptomatic. Elevated prolactin levels without any clinically relevant effects are more common in males, whereas women tend to present with significantly higher prolactin levels resulting in menstrual abnormalities and other adverse effects [1]. Clinical manifestations correlate with the extent of hyperprolactinemia. Some symptoms are due to hypogonadism caused by prolactin, which disturbs hypothalamic–pituitary axis function, while other symptoms are the result of direct effects on target tissues. Consequently, patients can suffer from sexual dysfunction, infertility, amenorrhea, gynecomastia, and galactorrhea. Data suggest that these symptoms are common, but patients rarely report them spontaneously so that clinicians underestimate their prevalence [4]. Hyperprolactinemia also has long-term consequences such as decreased bone mineral density and breast cancer [5]. Due to the delayed onset of these manifestations, they may mistakenly not be attributed to drug-induced hyperprolactinemia.

Galactorrhea secondary to prolactin elevation is more common in women than in men. Estimates of the prevalence of galactorrhea in cases of elevated prolactin levels vary from 10 to 50%. A well-conducted study found that 28/150 women (19%) developed galactorrhea within 75 days of commencing treatment with conventional APDs [6]*.* Only 8 women reported the symptom spontaneously to their treating physician. Lactation in nonpregnant women most likely causes distress in patients suffering from reality distortion and may also interfere with social and intimate relationships [7]*.* These facts illustrate the urgent need for a standardized survey regarding these severe adverse drug reactions (ADRs).

AMSP (AMSP = Arzneimittelsicherheit in der Psychiatrie = drug safety in psychiatry) is a continuous drug surveillance program in psychiatry that permits pharmacovigilance in a naturalistic setting. It is especially designed to evaluate severe adverse reactions to psychotropic drugs in psychiatric inpatients. AMSP generates an ongoing database of severe ADRs occurring in inpatients within psychiatric hospitals in Germany, Austria, and Switzerland.

This study presents cases of galactorrhea related to one or more antipsychotic drugs in women. The relative frequencies of the examined ADRs were estimated for selected compounds. This manuscript provides an update to AMSP’s publication “Galactorrhea Due to Psychotropic Drugs” from 2004 [8].

## Methods

Data on severe ADRs and psychotropic drug utilization have been collected by the European drug surveillance program “Arzneimittelsicherheit in der Psychiatrie” (AMSP) since 1993. This study includes data from 1993 to 2015. AMSP assesses severe ADRs [9] in the naturalistic setting of routine clinical treatment. Trained psychiatrists act as drug monitors collecting data on ADRs and documenting these cases using a standardized questionnaire. After review by a senior member of AMSP, the cases are discussed at central case conferences in which drug monitors from participating clinics gather alongside representatives of the Federal Health Agency (BfArM) and the Drug Commission of the German Medical Association (AkdÄ), as well as drug safety experts from the pharmaceutical industry. Here, the final judgment on the imputation of one or more drugs concerning the observed ADR is made including a probability rating of each drug assumed to be involved in the ADR [9].

- *Grade 1*: possible (ADR unknown or alternative explanation likelier)
- *Grade 2*: probable (ADR known for drug in question and time course and dosage in accordance with previous experience; alternative explanation less probable)
- *Grade 3*: definite (the same as G*rade 2* with re-occurrence of the ADR after re-exposure to the drug in question)
- *Grade 4*: questionable or not sufficiently documented.

When an agreement has been reached and probability ratings have been given to the imputed drugs, the case questionnaires are sent to the relevant authorities and pharmaceutical companies and saved in a fully anonymized manner at the central database of the AMSP for future analysis.

In case of polypharmacy, multiple drugs are often imputed. When a pharmacodynamic interaction is held responsible for an ADR, each of the imputed drugs is given a rating of “possible”, “probable”, or “definite” according to the given facts. In this report, we only refer to ADRs (grade 2 and 3) in which involvement of the drug(s) in question has been rated as “probable” or “definite”.

The AMSP database evaluates cases of ADRs from two different perspectives. The first perspective considers all events of an ADR in which one or more drugs were causally involved, therefore, also including ADRs associated with polypharmacy (referred to as “all cases”). The second perspective only focuses on events of an ADR, in which a single drug/drug class was causally involved (referred to as “imputed alone”).

The definition of a clinically severe ADR is given in a detailed study protocol [9]. As to the ADRs discussed in this paper, each case of “marked” galactorrhea (i.e., accompanied by distressing symptoms such as distinct pain or tension, enlargement of breasts with weight gain or soaked clothing with the need for pads) is defined as a “severe” ADR. Hyperprolactinemia without further sequelae is not rated as “severe”.

Data on drug use at the participating hospitals are assessed on two reference days per year on which all administered drugs and their doses are documented for all psychiatric inpatients along with basic demographic and diagnostic data. Moreover, the contributing hospitals provide the number of inpatients and the mean duration of inpatient care for all patients under surveillance per year.

### Ethics review

Evaluations based on the AMSP database have been approved by the Ethics Committee of the University of Munich and the Ethics Committee of the Hannover Medical School (Nr. 8100_BO_S_2018). This study adheres to the Declaration of Helsinki and its later amendments. The AMSP program is a continuous observational post-marketing drug surveillance program and does not interfere with the ongoing clinical treatment of the patients under surveillance. Furthermore, evaluation data were obtained from the anonymized data bank and individual patients cannot be traced.

### Statistical analysis

The incidence of galactorrhea was calculated in relation to the number of female patients exposed to a given compound, drug class, or subclass, respectively, and is reported with its 95% confidence interval (CI). Due to the low rate of galactorrhea, only drugs with 3000 or more patients exposed were included in statistical analysis for mandatory evaluation. With regard to the very low rate of serious side effects, such as severe galactorrhea, and the high number of individuals exposed, confidence intervals were calculated using the exact method (Vollset SE. 1993. Confidence intervals for a binomial proportion. Stat. Med. 12:809–824), avoiding the bias of commonly used approximate methods (Agresti A, Coull BA. Approximate is better than “exact” for interval estimation of binomial proportions. Am Stat 1998; 52: 119–126). Significance level was set at *p* < 0.05. Statistical comparisons of galactorrhea rates related to diagnoses, gender, and age were performed by means of Chi-square tests. Rates were calculated for established diagnostic groups and for age groups the following intervals were chosen: 16–30 years, 31–40 years, 41–55 years and > 55 years (postmenopausal).

## Results

### Sociodemographic and illness-related data

Between 1993 and 2015, a total of 475,096 psychiatric inpatients were monitored within the AMSP program in 86 hospitals. During this time frame, 175,884 women (320,383 patients in total) were treated with APDs for the main indications of schizophrenia, schizotypal, and delusional disorders, as well as mood and organic disorders. Galactorrhea was assessed as a severe ADR during antipsychotic treatment in 170 cases. This corresponds to a rate of 0.97 cases in 1000 female inpatient admissions. No cases were assessed in males.

The occurrence of galactorrhea presented with highly significant differences among age groups (Table 1; *p* < 0.0001). Women between 16 and 40 years of age were most commonly affected, with 80% of all cases occurring within the reproductive age and an incidence of 2.57 in 1000 inpatients. The rate was highest in the youngest group between 16 and 30 years (3.18 in 1000 inpatients). A total of 31 cases were identified among patients between 41 to 55 years (18% of all cases, incidence 0.59 in 1000 inpatients), while patients aged 55 years and older reported only 3 cases (2% of all cases, incidence 0.04 in 1000 inpatients). Cases of galactorrhea occurred significantly more frequently (*p* < 0.0001) in patients treated with APDs diagnosed with schizophrenia, schizotypal, and delusional disorders (1.34 per 1000), organic mental disorders (1.27 per 1000), as well as mania (1.04 per 1000). Patients treated with APDs suffering from unipolar depression (0.62 per 1000) or neurosis and personality disorders (0.53 per 1000; see Table 1) were much less commonly affected.

**Table 1:** ICD-10 diagnosis and age and monitored female inpatients exposed to antipsychotics (*N* = 175.884) compared to ADR cases with galactorrhea (*N* = 170)

	Monitored female inpatients with APDs, *n* (% of 175,884)	Cases with galactorrhea, *n* (% of 170)	Incidence in ‰ inpatient admissions	*P* value
**Diagnosis (ICD-10)**				*p* ≤ 0.0001*
Schizophrenia, schizotypal, and delusional disorders (F2−)	73,352 (42)	98 (58)	1,34	
Depression (F31/F32/F33.−)	52,964 (30)	33 (19)	0,62	
Mania (F30/ F31.-)	5746 (3)	6 (4)	1,04	
Organic mental disorders (F0−)	17,295 (10)	22 (13)	1,27	
Neurosis/personality disorders (F4−/ F6−)	20,708 (12)	11 (6)	0,53	
“Others” (F1−/F5−/F7−)	5819 (3)	0 (0)		
**Age**				*p* ≤ 0.0001**
16–30	24,858 (14)	79 (46)	3,18	
31–40	28,087 (16)	57 (34)	2,03	
41–55	52,339 (30)	31 (18)	0,59	
> 55	70,600 (40)	3 (2)	0,04	

*χ2 = 28.24, *df* = 5, *p* = 3.2623E−05

**χ2 = 228.79, *df* = 3, *p* = 2,5316E−49

### Antipsychotic drugs associated with galactorrhea

Seventeen different APDs were attributed to 170 cases of galactorrhea. In 132 patients (77.4% of all galactorrhea cases), a single antipsychotic drug was held responsible for the ADR as the only probable cause (rated as grade 2). Only 20 of these cases (15.2%) occurred under monotherapy (three each under olanzapine and paliperidone, six under amisulpride, and eight under risperidone). In 76 cases (57.6%), another drug (between one and four substances per case) was imputed as possible contributor to galactorrhea (rated as grade 1). In 38 cases, combinations of several drugs were imputed as equal contributors to the galactorrhea (rating as grade 2). Most of cases imputing more than one drug were due to the combination of two antipsychotic drugs, however, the individual numbers are too small to identify a specific risk combination of APDs.

**Fig. 1:**
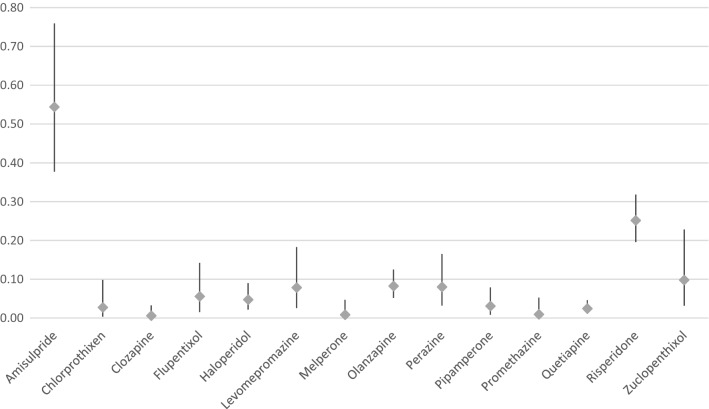
Occurrence rates (95% confidence intervals) of galactorrhea with imputed drugs

**Fig. 2:**
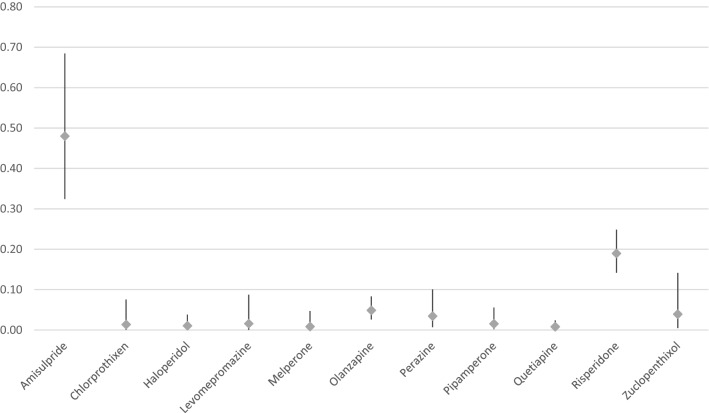
Occurrence rates (95% confidence intervals) of galactorrhea with imputed drugs (only imputed alone)

Table 2 as well as Figs. 1 and 2 show the rates of galactorrhea under treatment with different substances. Amisulpride showed the highest relative risk for inducing galactorrhea and was considered to have caused the ADR in 30 cases (0.48% of all patients exposed). Most cases occurred under treatment with risperidone which was imputed alone in 53 cases (0.19% of all patients exposed). Olanzapine was imputed alone as probable cause in 13 cases (0.05% of all patients exposed). Among subgroups of APDs, we found most cases of galactorrhea to arise under the treatment with second generation APDs which were consequently imputed alone in 119 cases or 0.11% of all patients exposed. In the class of first-generation APDs, most cases appeared under treatment with high potency APDs which were imputed alone in 17 cases or 0.04% of all patients exposed. Regarding the homogenous chemical subgroups of first-generation APDs, most cases of galactorrhea occurred under treatment with thioxanthenes (imputed alone in 7 cases or 0.04% of all patients exposed). Other drugs were imputed only as a possible additional contributor to galactorrhea (e.g. low potency APDs, selective serotonin reuptake inhibitors [SSRI], selective serotonin and noradrenaline reuptake inhibitors [SSRNI]), when the time course was unusual or because these drugs only rarely lead to galactorrhea. Of the 30 cases in which amisulpride was imputed alone as a probable cause, other APDs or antidepressant drugs were imputed as possible contributors in 12 cases. Monotherapy with amisulpride was observed in only 6 cases of galactorrhea. Of the 53 risperidone cases, where the substance was imputed alone as a probable cause, risperidone was prescribed as monotherapy in only 8 cases. In 22 cases, other APDs or antidepressant drugs were imputed as possible contributors. In 5 of the 13 olanzapine cases, SSRIs or venlafaxine were additionally prescribed; in 1 case, olanzapine was combined with valproate and in 3 cases, with other APDs that were imputed as only possible contributors due to the course of time (twice pipamperone and once perazine). Only 2 cases of olanzapine as monotherapy (20 mg/d in both cases) were observed. Benzodiazepines, a common comedication, were not applied more frequently in galactorrhea cases overall (29.4%) in comparison to all patients exposed to APDs (33.7%). There were not enough patients exposed to long-acting injectables (LAI) to perform a separate analysis. Flupentixol LAI was imputed alone in 2 cases, paliperidone and risperidone LAI in one case each. Zuclopenthixol LAI was imputed in combination therapy in one case, but was not imputed alone. Galactorrhea in relation to aripiprazole was not observed. Clozapine was imputed in combination therapy in 1 case, but never imputed alone. Due to the low prescription rates of paliperidone over the period of our survey, it was not considered for further analysis. Of the 1367 patients treated with paliperidone, 12 experienced clinically severe galactorrhea, in 10 cases, paliperidone was imputed alone as probable cause (1 case under application of the depot, 9 cases under oral medication).

**Table 2:** Frequency of galactorrhea between 1993 and 2015 under APDs in relation to all patients exposed (n > 3000)

Antipsychotic drug	Patients exposed, *n*	Drug group/single APDs imputed alone and in combination, *n* (%)	Drug group/single APDs imputed alone, *n* (%)
**First Generation APD**	92,829	44 (0.05)	22 (0.02)
High Potency APD	46,850	34 (0.07)	17 (0.04)
Low Potency APD	57,448	13 (0.02)	5 (0.01)
*Butyrophenones*	45,508	16 (0.04)	5 ( 0.01)
Haloperidol (incl. depot)	20,108	9 (0.05)	2 (0.01)
Melperone	11,868	1 (0.01)	1 (0.01)
Pipamperone	12,987	4 (0.03)	2 (0.02)
*Thioxanthenes*	18,918	14 (0.07)	6(0.03)
Chlorprothixen	7361	2 (0.03)	1 (0.01)
Flupentixol (incl. depot)	7201	6 (0.08)	2 (0.03)
Zuclopenthixol (incl. depot)	5107	7 (0.14)	3 (0.06)
*Phenothiazine*	38,882	14 (0.04)	5 (0.01)
Levomepromazine	6378	5 (0.08)	1 (0.02)
Perazine	8727	7 (0.08)	3 (0.03)
Promethazine	10,590	1 (0.01)	0 (0)
Prothipendyl	9159	0 (0)	0 (0)
**Second generation APD**	115,703	144 (0.13)	119 (0.11)
Amisulpride	6250	34 (0.54)	30 (0.48)
Aripiprazole (incl. depot)	7808	0 (0)	0 (0)
Clozapine	17,015	1 (0.01)	0 (0)
Olanzapine (incl. depot)	26,734	22 (0.08)	13 (0.05)
Quetiapine	37,012	9 (0.02)	3 (0.01)
Risperidone (incl. depot)	27,437	69 (0.25)	53 (0.19)

In 18 cases, APDs used in less than 3000 patients were imputed alone: paliperidone 9 cases,—palmitate 1 case, sulpiride 4 cases, ziprasidone 3 cases, fluphenazine 1 case

Among 170 cases of galactorrhea, 3 cases were attributed to quetiapine alone as a probable cause. This finding is significant insofar that quetiapine has only rarely been associated with elevated prolactin levels and galactorrhea in the currently available literature. All 3 cases of quetiapine-associated galactorrhea affected patients suffering from mood disorders. In the first case of a 48-year-old woman with severe mania, galactorrhea started after 1 month of treatment with quetiapine 600 mg/d. The serum level of quetiapine was elevated (996 nmol/l, ref. 80–780 nmol/l). Zuclopenthixol 6–25 mg had been given in addition to quetiapine for 2 weeks, but discontinued the day before the galactorrhea started. Galactorrhea persisted for 3 weeks and disappeared 1 week after discontinuation of quetiapine. Therefore, zuclopenthixol was rated as only a possible contributor to the ADR. The medication was switched to risperidone 4 mg/d in monotherapy, under which galactorrhea did not re-appear despite further increases in prolactin levels. In the second case of a 30-year-old woman with a manic episode with psychotic symptoms, galactorrhea was present after 1 week of treatment with up to 800 mg quetiapine per day. In addition, the patient was treated with 1125 mg of lithium. Levomepromazine (given in a maximum dose of 100 mg per day for 4 weeks) had been discontinued the day prior to the occurrence of galactorrhea. As in the previous case, levomepromazine was rated as only a possible contributor to the ADR due to the time course. In this case, elevated prolactin levels could not be detected. Quetiapin was tapered over 16 days while continuing treatment with lithium, causing the symptoms to mitigate and finally dissolve completely. In the third case of a 64-year-old female patient suffering from a major depression with psychotic symptoms, galactorrhea presented under treatment with 400 mg quetiapine per day. Quetiapine was started 10 weeks before the occurrence of galactorrhea with 400 mg, then 200 mg. Afterwards, dose was again increased to 400 mg for 3 weeks, during which time treatment with venlafaxine extended release 75–150 mg was additionally started. Another possibly contributing factor in this case was the second and last intramuscular injection of risperidone depot 25 mg 6 weeks prior to the beginning of the ADR. The prolactin level was 103.3 ng/ml 1 day after galactorrhea had started (ref. < 15 ng/ml). Quetiapine was tapered and finally discontinued over the course of 2 weeks with galactorrhea stopping 9 days later under continuation of treatment with venlafaxine. Venlafaxine and risperidone were rated only as possibly contributing factors due to time course.

For the sake of completeness, we want to mention 5 cases of galactorrhea in our database that were not related to antipsychotic drugs. In one case, pregabalin (300 mg/day) was imputed as probably responsible in a combination therapy with carbamazepine, prothipendyl, and trazodone. Galactorrhea stopped after discontinuing pregabalin, while all other substances were continued and partly even increased. 2 cases were reported under monotherapy with antidepressant drugs (trimipramine and paroxetine), 1 case under a combination of trimipramine and paroxetine*,* and 1 case under the combination of imipramine and doxepin.

### Drug dosages

Table 3 gives information on median daily dosage of patients suffering from galactorrhea and all exposed patients. For most of the drugs, the daily dosages were higher among patients with galactorrhea compared to all patients exposed; exceptions were mainly amisulpride and zuclopenthixol.

**Table 3:** Median daily dosages in monitored patients and galactorrhea cases under treatment with imputed antipsychotic drugs*

Antipsychotic drug	Median dosage (mg/day), all patients exposed (min./max.)	Median dosage (mg/day) all cases (min./max.)	Median dosage (mg/d) imputed alone (min./max.)
Amisulpride	400 (50/1200)	400 (150/1200)	400 (150/1200)
Olanzapine	10 (1,25/60)	20 (5/50)	20 (10/40)
Perazine	200 (25/800)	250 (75/700)	200 (100/700)
Quetiapine	200 (12,5/1800)	600 (200/1000)	600 (400/800)
Risperidone (oral only)	2 (0.25/10)	4 (0.5/8)	4 (0.5/8)

*Only drugs with at least 3 or more cases (imputed alone)

The administered dosages in all exposed patients differed in relation to the diagnoses for the 3 drugs most often involved in galactorrhea: risperidone, amisulpride, and olanzapine.

Median daily dose of oral risperidone was 4 mg in patients with schizophrenia, 2 mg in patients with depression, 3 mg in patients with mania, and 1 mg in patients with organic mental disorders. The median daily dosages were identical in the group of galactorrhea cases except in patients with organic mental disorders (median daily dosage 3.5 mg). Among all 67 (52 imputed alone = i.a.) cases of risperidone-induced galactorrhea, the daily dosage was > 2 mg in 42 cases (34 i.a.), between 1.1 and 2 mg in 16 (10 i.a.) cases, and 1 mg or less in 9 (8 i.a.) cases. There were only 2 cases under risperidone depot (1 case imputed alone under 50 mg every 2 weeks) so the dosages mentioned above refer to the oral medication, that was imputed alone in 52 cases.

Patients suffering from schizophrenia were prescribed a median daily dosage of 600 mg amisulpride, while patients with depression were treated with 300 mg/d, and patients with mania as well as organic mental disorders were prescribed 400 mg/day. In cases of galactorrhea causally associated with amisulpride, the median daily dosage was 400 mg for all diagnoses except for patients suffering from schizophrenia, who were prescribed a median dosage of 500 mg per day. Among 29 of all cases in which amisulpride was imputed, galactorrhea occurred under a daily dosage of min. 200 mg (median 400 mg), in 5 cases the dosage was between 150 and 200 mg per day.

Among users of olanzapine, the median dosage per day was 15 mg in patients with schizophrenia and mania, 10 mg in patients with depression, and 7.5 mg in patients with organic mental disorders. The median daily dosage of olanzapine was higher among patients with galactorrhea than in the group of all patients exposed for the diagnoses schizophrenia (20 mg) and organic mental disorders (15 mg). The dosage in patients with depression was the same (10 mg), while olanzapine-induced galactorrhea was not recorded in patients with mania. Of the 13 cases in which olanzapine was imputed alone, 4 occurred under a daily dosage of 10 mg, 2 cases under 15 mg, 5 cases under 20 mg, and 1 case each under 30 resp. 40 mg.

### Prolactin values

Serum prolactin levels were documented in 123 (72.4%) of the 170 galactorrhea cases, the reference value was set to 15 ng/ml. The mean value was 110.75 ng/ml (median value 91.76 ng/ml; min. 6.80, max. 430.50 ng/ml). Cases in which multiple prolactin levels were given, the highest value was used for further analysis. In our sample, we found 5 cases (2.9%) of galactorrhea in spite of normal levels of prolactin. The drugs imputed were amisulpride (with a daily dosage of 300 mg), risperidone (3 mg/d), olanzapine (10 mg/d), haloperidol (10 mg/d), and quetiapine (800 mg/d) in 1 case each.

### Countermeasures and course of the ADR

Discontinuation of the implicated drug(s) was performed in 145 (85.3%) of the 170 patients with galactorrhea. In 20 other patients (11.8%), dosage of the imputed drug was reduced, whereas no changes in medication were made in the remaining 5 patients (2.9%). Further consultation by an internal medicine specialist or gynecologist was provided in 20 cases (11.8%). In only 6 cases, drugs to counteract galactorrhea were used (3 times additional treatment with bromocriptine and one time each with cabergoline, metergoline, and calcium + magnesium). Galactorrhea disappeared in 127 cases (74.7% of all cases), mostly after discontinuation or dose reduction of the implicated drug. In 1 case, symptoms ceased with no changes in medication, while in another case, symptoms receded following the addition of bromocriptine. In 26 patients (15.3%), galactorrhea improved but was still present at the end of the observation period. In 14 patients, galactorrhea was unchanged at the end of observation whereas the further course was unknown in 3 patients. Further contributory risk factors in addition to those mentioned above (female gender, age, drug dosage) could not be identified.

## Discussion

Hyperprolactinemia and galactorrhea are common phenomena under treatment with antipsychotic drugs. Risk factors for antipsychotic-induced hyperprolactinemia which have also been described by other studies [10] include adolescence, high dose of APDs, and female sex, particularly in the reproductive age*.* We could not identify any cases of galactorrhea in men and most cases were assessed in women between 16 and 30 years of age suffering from schizophrenia, a clinical indication for which higher dosages of the antipsychotic drugs were used than within treatment of the other diagnoses.

Most cases of galactorrhea occurred under treatment with risperidone while amisulpride had the highest relative risk for galactorrhea. The high calculatory risk for galactorrhea under treatment with paliperidone is not yet statistically applicable due to low case counts, but should be observated in clinical practice. Despite solid evidence that amisulpride-induced hyperprolactinemia does not seem to be strongly dose-related and has been reported to occur under treatment with only 50 mg/day in the literature [11–13], we did not document any cases in which the patients were treated with a daily dosage of 150 mg or less.

A possible additional effect of different drugs has to be discussed in many of the AMSP cases, but especially in the cases, where olanzapine, which rarely leads to galactorrhea, was imputed alone. In our study, we found a number of substances, e.g. SSRIs, SSNRIs, tricyclic antidepressant drugs, other APDs, benzodiazepines, and mood stabilizers to have possibly contributed to galactorrhea, especially in combination therapy. A metaanalysis by Egberts et al. [14] shows that serotonergic antidepressant drugs are associated with an approximately eight times higher risk of non-puerperal lactation compared to non-serotonergic antidepressant drugs. Literature reviews suggest that nearly 95% of case reports of galactorrhea under treatment with antidepressant drugs are attributed to SSRIs, mainly paroxetine, fluoxetine, sertraline, fluvoxamine, and escitalopram [15, 16]*.* In our sample, we did not observe a case due to SSRIs alone, but three cases related to tricyclic antidepressants were documented, one of which has previously been published as a case report [17]*.* So far, valproate has not been linked to increasing prolactin levels, whereas the data on benzodiazepines is inconsistent [18]. Benzodiazepines have potent effects on inhibition of prolactin secretion in response to stressful and pharmacological stimuli and usually, in common dosages, reduce the hormonal response, possibly through a direct action on the anterior pituitary gland [19, 20]*.* While Laakmann found no changes in the secretion of prolactin under treatment with benzodiazepines in healthy and depressed adults while resting [21], Zemishlany et al. [22] identified a robust increase in prolactin levels in response to the γ-aminobutyric acid (GABA) agonist alprazolam, a finding which is not consistent with previous data on traditional benzodiazepines. They reported that plasma prolactin levels increased by 100% 2–8 h after a single dose (3 mg) of alprazolam, further reporting a case of galactorrhea under alprazolam in monotherapy in very high dosage (9 mg per day) [23]. This may indicate an at least additional role of substances such as antidepressant drugs and benzodiazepines in the emergence of galactorrhea. The effects of lithium on prolactin secretion are discussed controversially and probably involve both dopamine and serotonin pathways [18]*.* A case report previously described the occurrence of galactorrhea in a female patient under monotherapy with lithium carbonate [24]*.*

We want to highlight these findings and enhance awareness for the different substances playing a role in substance-induced galactorrhea, not only the hereof well-known antipsychotics.

Besides occurring under treatment with high-risk APDs such as risperidone and amisulpride, galactorrhea is also to be expected in patients treated with APDs thought to have a lower risk for this ADR. For example, we identified three cases of galactorrhea related to quetiapine, which has been recommended as an alternative in patients who have previously developed galactorrhea associated with other APDs. Since quetiapine rarely leads to hyperprolactinemia and galactorrhea, there are only a few cases presented in the literature [25] and the risk for this ADR is still low compared to other APDs. Clinicians should be aware of the possible occurrence of galactorrhea under treatment with quetiapine, especially when combined with another prolactin-raising drug*.*

As in the current literature, our sample did not include any cases of aripiprazole-induced galactorrhea. Aripiprazole or other prolactin-sparing atypical APDs like cariprazine may be an alternative treatment option or be considered as adjunctive therapy in some cases of psychotropic-induced hyperprolactinemia [26]. The S3 guidelines on schizophrenia also lists add-on low-dose aripiprazole as a treatment option for hyperprolactinemia related to APDs [27]. However, there are cases, where aripiprazole is not effective to stop hyperprolactinemia/galactorrhea, e.g. when induced by amisulpride [28].

In our sample, we found 5 cases of galactorrhea without elevated levels of prolactin. Although prolactin elevation is usually mild (25–100 ng/mL), in cases of drug-induced hyperprolactinemia, it is also highly variable [29]. Artificially low prolactin levels may result from the so-called “hook effect”, which is an assay artifact caused by an extremely high level of prolactin, which saturates the detecting antibody used in the prolactin assay, thus, resulting in a falsely low value [30]*.* As symptoms of galactorrhea usually persist longer than hyperprolactinemia, in some cases, prolactin levels may have been measured after having already decreased, therefore, explaining the detection of normal values in our sample. This can be explained by the physiological effects of prolactin on the development of the female mammary gland [31]*.* When prolactin levels normalize after discontinuation of the imputed drug, the glandular tissue may take more time to adjust. Another possible explanation could be the combination with serotonergic drugs in four of these cases (clomipramine, fluvoxamine, escitalopram, paroxetine, and citalopram) and lithium in one case. There are a few case reports of documented euprolactinemic galactorrhea relating to SSRIs, even without additional antipsychotic drugs [32–34] and even one case with quetiapine in monotherapy [25]*.*

Since the spectrum of indications for APDs is constantly growing and APDs are frequently used “off-label” in patients without delusional symptoms, the importance of galactorrhea in clinical practice is increasing. By enhancing awareness of adverse drug reactions, we may be able to identify or perhaps even prevent these in a timelier manner, sparing patients a great deal of trouble.

## Limitations

In the course of clinical trials, ADRs are described in a limited and defined population, while unstructured reporting systems have the problem of incompleteness and unreliability. In contrast to prospective, placebo-controlled studies under a randomized design with healthy controls, the data obtained in this naturalistic setting have several limitations. Underreporting has to be taken into account, so that the incidence rates of galactorrhea may be underestimated. The reporting of severe events depends on clinicians acting as individual drug monitors during routine work in the ward. Depending on their time and motivation as well as the financial means of the participating hospital, an individual and institutional bias in terms of underreported ADRs cannot be ruled out [35]*.* In addition, clinical practice and literature both show that only part of the afflicted women report this distressing event spontaneously [7]*.*

Another potential cause of the low rates of the ADR is the strict definition within AMSP regulations, in which only severe symptoms are considered. In addition, we only recorded cases of galactorrhea presenting during inpatient care, while data regarding long-term treatment or complications resulting from hyperprolactinemia were not available.

Another problem are the inconsistent measures, especially regarding blood work (drug monitoring and prolactin levels), since these values are not yet part of clinical routine. In some cases, laboratory values were not available and even if blood work had been done, values did not necessarily represent the same situation (max. levels vs. no defined point of time in the course of the ADR), so the comparability of these values is limited.

Since polypharmacy and the imputation of more than one drug were included, the assessment of correlation and probability rating can be more difficult in some cases and may be more susceptible to errors.

On the other hand, in contrast to case–control studies, the AMSP data bank allows statistical analyses for the occurrence rates and risk of an ADR under a given medication under “real-life” circumstances. Incidence rates of ADRs are compared to the overall prescription rates of psychotropic drugs of which data are gathered on two reference days per year.

## Conclusion

Overall galactorrhea is a severe and probably underestimated condition among patients treated with psychotropic, especially antipsychotic, drugs. Most of the application data in our naturalistic setting are in agreement with the currently available literature. However, a few unusual cases highlight the importance of alertness in clinical practice and the importance of taking a patient’s individual situation into consideration.
